# Evaluation of Comorbidities and Treatment Outcome in Various Subtypes of Lichen Planus: A Single-Center Retrospective Study

**DOI:** 10.3390/jcm15114101

**Published:** 2026-05-26

**Authors:** Ken Goekcimen, Cadri Knoch, Fabienne Fröhlich, Thomas Kuendig, Christian Greis, Barbara Meier-Schiesser

**Affiliations:** Department of Dermatology, University Hospital of Zurich, Rämistrasse 100, 8091 Zurich, Switzerland; kensuemer.goekcimen@uzh.ch (K.G.); cadri.knoch@usz.ch (C.K.); fabienne.froehlich@usz.ch (F.F.); thomas.kuendig@usz.ch (T.K.);

**Keywords:** lichen planus, lichen planopilaris, epidemiology, comorbidity, health register data, treatment

## Abstract

**Background**: Lichen planus (LP) is a chronic inflammatory dermatosis with multiple clinical variants involving the skin, mucous membranes, nails, and hair follicles. **Methods**: We conducted a retrospective, descriptive study of patients diagnosed with LP at a tertiary referral center from January 2011 to December 2024. Inclusion required concordance between clinical presentation and histopathologic findings. Demographic characteristics, LP subtypes, anatomical involvement, comorbidities, therapeutic approaches, and treatment outcomes were extracted from electronic health records. In addition, an exploratory sensitivity analysis restricted to patients with a single LP subtype was performed to allow for independent subgroup comparisons, and a modified Charlson Comorbidity Index (CCI) based on available comorbidity domains was calculated. Pairwise multivariable logistic regression models adjusted for age, sex, and outcome-specific modified CCI were performed for selected comorbidities. **Results**: A total of 754 patients were included (mean age 53.1 years), with cutaneous LP (cLP), oral LP (oLP), genital LP (gLP), and lichen planopilaris (LPP) being the most frequent subtypes; a total of 620 had a single major LP subtype and were included in the mutually exclusive analysis. In these groups, modified CCI, age-adjusted modified CCI, and overall comorbidity count differed significantly across subtypes (Kruskal–Wallis *p* < 0.001). After adjustment in pairwise models, cLP-only showed higher odds of malignancy compared with oLP-only, gLP-only, and LPP-only and higher odds of diabetes mellitus compared with all other pure subtypes. Most other comorbidity comparisons were non-significant or imprecise because of low event numbers. Topical glucocorticoids were the most frequently used treatment, and treatment responses varied by subtype, being more effective in cLP and gLP compared to LPP. Topical calcineurin inhibitors demonstrated the highest response rates in gLP. Acitretin was most effective in cLP, whereas isotretinoin showed favorable responses in oLP. **Conclusions**: This large, histopathologically confirmed cohort highlights distinct differences in comorbidity patterns, anatomical involvement, and therapeutic response across LP subtypes. Treatment outcomes vary substantially by subtype, underscoring the need for individualized management strategies. Prospective studies are warranted to further elucidate subtype-specific disease associations and optimize treatment approaches.

## 1. Introduction

Lichen planus (LP) is a chronic inflammatory disease that can affect the skin, mucous membranes—particularly of the oral and genital regions—as well as the nails [1] and the hair follicles [2]. The estimated global prevalence of LP ranges from 0.2% to 1%, with the condition most commonly affecting middle-aged individuals [3,4]. In contrast, cutaneous LP (cLP) rarely occurs in children [5]. The follicular form is more frequent in women than in men, with a female-to-male ratio of approximately 9:1 [6].

Depending on the morphology and localization of the lesions, LP manifests in different subtypes, such as cLP, oral mucosal LP (oLP), genital mucosal LP (gLP), lichen planopilaris (LPP), nail LP and other rare variants [7], and can affect several skin sites, either simultaneously or successively [8,9]. LP is further classified according to clinical appearance such as eruptive/exanthematous LP, hypertrophic LP and bullous LP [9]. In about half of the patients, the lesions occur simultaneously on the skin and the oral mucosa [10].

The clinical presentation of LP varies according to the body sites involved, but the disease exhibits a uniform histological phenotype. In most cases, the diagnosis is made clinically, particularly when lesions occur at predilection sites such as the forearms or legs [11]; however, a skin biopsy is often performed to confirm the diagnosis. In oLP, biopsies are essential to exclude malignant transformation [12,13,14].

Similar to other inflammatory dermatoses, immune dysregulation is thought to play a pivotal role in the pathophysiology of LP. Evidence indicates that activated T lymphocytes, particularly cytotoxic CD8^+^ T cells, mediate a cell-directed immune response against basal keratinocytes, leading to apoptosis. This process is amplified by CD4^+^ helper T cells through the secretion of Th1-associated cytokines, including interferon-γ (IFN-γ), tumor necrosis factor-α (TNF-α), and interleukin-2 (IL-2), which further promote keratinocyte apoptosis and perpetuate local inflammation [15]. However, the exact pathomechanisms remain incompletely understood. Proposed mechanisms linking LP to systemic comorbidities include chronic Th1-skewed inflammation, epithelial barrier dysfunction, immune cross-reactivity, and, in selected cases, medication-induced lichenoid reactions. These mechanisms may differ between cutaneous, mucosal, and follicular subtypes and could contribute to subtype-specific comorbidity profiles.

Associations of LP with viral infections have been reported, with LP appearing more frequently in patients with hepatitis B or C infections [16,17,18], although several studies challenge this causal relationship [19,20,21,22].

The likelihood of oLP progressing to squamous cell carcinoma is between 0.34 and 0.69% per year [23]. gLP—particularly the erosive vulvar form—has been associated with a small but documented risk of squamous cell carcinoma; however, the current data do not allow for definitive conclusions [24,25]. cLP is not considered to be associated with cancer [26].

Associations between LP and various autoimmune conditions, such as alopecia areata [27], autoimmune thyroiditis [28], celiac disease [29], dermatomyositis [30], type 1 diabetes mellitus [30], Sjogren’s syndrome [30], and systemic lupus erythematosus [31], have been reported. Additionally, LP has been linked to psychological comorbidities, including anxiety and depression [32]. One limitation in the evaluation of treatment outcomes is that some patients received multiple therapeutic agents, which limits the ability to attribute clinical response to a single intervention. Another limitation is that concomitant medications used to treat comorbidities were not systematically recorded for analysis. This is relevant because drug-induced lichenoid eruptions and possibly LPP-like reactions may mimic or modify the clinical phenotype, thereby introducing potential confounding or misclassification.

Topical glucocorticoids are typically the cornerstone of treatment for localized LP [3,15,33]. If there is no improvement with topical therapy, or in cases of acute exacerbation or widespread disease, systemic glucocorticoids may be administered [34]. Second-line therapy such as phototherapy [4,15], oral retinoids [35,36], hydroxychloroquine [37,38], and cyclosporin [39] can be considered for persistent cases. Other immunosuppressive agents such as sulfasalazine [40], methotrexate [41] and azathioprine [42], and immunomodulators such as thalidomide [42], adalimumab [43], JAK inhibitors [44] and IL-17 inhibitors [45] have been applied successfully in cutaneous LP. However, these treatments are generally reserved for refractory cases due to limited evidence, high cost, or unfavorable side effect profiles [11].

The aim of this study was to evaluate the characteristics of patients with LP and its subtypes treated and followed at our institution, as well as to compare the different LP subtypes with respect to comorbidities, therapeutic approaches, and clinical presentation.

## 2. Materials and Methods

### 2.1. Patient Cohort

Histopathologic reports were retrieved from our hospital’s internal database, DermaPro (ifms—Institut für medizinische Software). All tissue samples were evaluated by board-certified dermatopathologists or pathologists. Histopathologic findings were systematically compared with clinical presentations by reviewing corresponding reports and images from the electronic patient records. Inclusion criteria for LP required concordance between the clinical presentation and the histopathologic diagnosis. Exclusion criteria included incomplete clinical or histopathologic data, uncertain diagnoses, and cases with insufficient documentation in the patient records. A total of 754 patients met these criteria. All patients were seen at the outpatient clinics of the Department of Dermatology, the Department of Immunology, the Women’s Clinic, the Department of Otorhinolaryngology, Head and Neck Surgery, or the Department of Oral and Maxillofacial Surgery at the University Hospital of Zurich between January 2011 and December 2024. Written informed consent was obtained from all patients, and the study was approved by the Ethics Committee of the Canton of Zurich (KEK 2021-00958).

### 2.2. Data Analysis

Data were collected for the following variables: sex, age, comorbidities, therapies, treatment outcomes, anatomical sites of involvement, and LP subtypes. Comorbidities known to be associated with LP, as reported in the literature (21, 46, 47), as well as treatments used for LP, were extracted from the electronic health records. The prevalence of predefined comorbidities was assessed based on confirmed outpatient diagnoses and principal or secondary hospital discharge diagnoses, defined according to ICD-10-GM codes (2018). Although therapies used for LP were extracted systematically, concomitant medications prescribed for comorbid conditions were not analyzed in a standardized manner. Therefore, potential drug-induced lichenoid or lichen planopilaris-like reactions could not be reliably assessed in this cohort. Information on disease activity, severity, or treatment control of comorbidities was not systematically available in the retrospective electronic health records and was therefore not included in the analysis. Malignant diseases were categorized by differentiating non-melanoma skin cancer (NMSC) from other malignancies, including solid tumors and hematological cancers. Treatment response was stratified into four categories: 0 = no response or clinical deterioration, 1 = stable disease, 2 = marked improvement or complete remission as documented by the treating physician (reflecting clear clinical improvement of lesions and/or symptoms), and 3 = loss to follow-up.

Prevalence estimates for comorbidities, therapies, and clinical presentations were calculated as the proportion of affected patients relative to the total cohort of LP. Because the main LP subtype groups were overlapping and therefore not statistically independent, the primary full-cohort analysis remained descriptive.

To address potential bias due to the overlapping clinical subtypes, we conducted an additional exploratory sensitivity analysis restricted to patients with a single major LP subtype only. Patients with the involvement of more than one of the four main subtypes (cLP, oLP, gLP, and LPP) were excluded from this analysis. Within this subset of mutually exclusive groups, continuous variables (age) were compared using the Kruskal–Wallis test. Categorical variables (sex and comorbidities) were compared using the chi-square test where appropriate. A modified Charlson Comorbidity Index (CCI) was calculated from available Charlson domains in the dataset: diabetes mellitus, liver disease, renal failure, connective tissue/rheumatologic disease, malignancy, and HIV infection. Because not all Charlson domains and severity levels were systematically available, this score is referred to as a modified CCI. An age-adjusted modified CCI was also calculated. Pairwise multivariable logistic regression models were fitted in the mutually exclusive subtype subset for selected comorbidities. Each pairwise model directly compared two pure subtype groups and adjusted for age, male sex, and modified CCI. To avoid circular adjustment, the modeled outcome was excluded from the modified CCI covariate where applicable. Results are presented as odds ratios (ORs) with 95% confidence intervals (CIs) and should be interpreted as exploratory.

## 3. Results

### 3.1. Patient Characteristics and Subtype Distribution

We identified a total of 754 patients. Patients had a mean age of 54.94 years (standard deviation [SD] 16.43) for cLP, 57.10 years (SD 15.7) for oLP, 53.41 years (SD 16.03) for gLP and 51.34 years (SD 16.81) for LPP and were predominantly female (cLP 53.9%, cLP 59.1%, LPP 83.2%), whereas gLP showed a male predominance (58.8%) (Table 1).

Overall, most manifestations showed a female predominance, including cLP (53.9%), oLP (59.1%), and, in particular, LPP (83.2%). In contrast, gLP demonstrated a male predominance (58.8%) in our cohort (Table 1).

**Figure 1:**
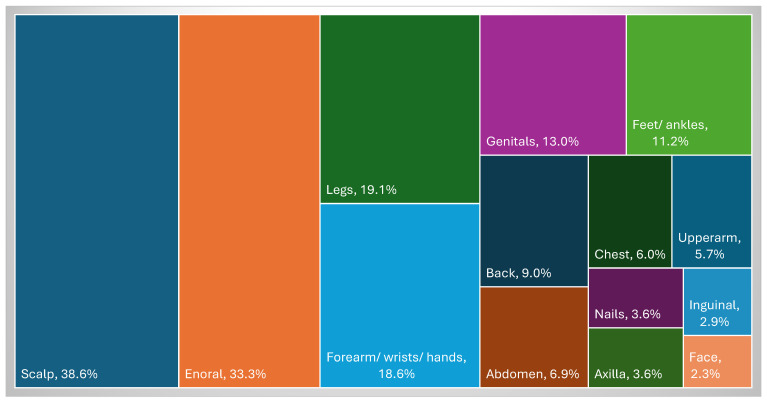
Tree map showing the skin localization (%) in 745 LP patients with LP. Note: Additional skin localizations not represented in the tree map included shoulder (1.6%), neck (1.6%), anal/perianal region (1.3%), gluteal region (1.2%), submammary area (0.8%), esophagus (0.5%), ocular region (0.1%), and nasal mucosa (0.1%).

Of the 754 patients included, 256 (34.0%) had cutaneous involvement, 251 (33.3%) had oral involvement, 97 (13.0%) had genital involvement, 29 (3.8%) had nail involvement, and 1 (0.1%) had ocular involvement. LPP was diagnosed in 291 patients (38.6%) (Table 1). The most common sites of cLP were the legs (52.7%) and the forearms, wrists, or hands (51.6%). In approximately 20% of oLP cases, the forearm/wrist/hands were also affected. In gLP, the oral cavity was involved in over 40% of cases. In contrast, LPP almost exclusively affects the scalp (see Figure 1 and Supplementary Figures S1–S4). On average, nearly three anatomical skin sites were affected in patients with cLP (mean: 2.97), compared with gLP (mean: 2.52), oLP (mean: 2.15), and LPP (mean: 1.31).

### 3.2. Overlap Between Clinical Subtypes

**Figure 2:**
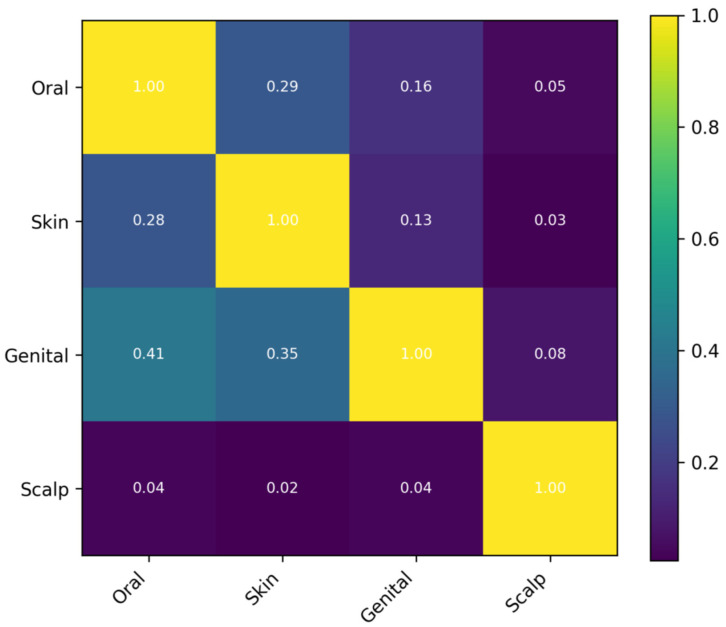
Pairwise Overlap of Clinical Lichen Planus Subtypes. A heatmap illustrating the pairwise co-occurrence of LP subtypes. Each cell represents the normalized proportion of patients with the subtype indicated on the *y*-axis who also exhibit the subtype on the *x*-axis. Warmer colors indicate higher overlap frequencies. Diagonal values (1.00) reflect complete overlap within each subtype.

The pairwise overlap between LP subtypes is illustrated in a heatmap (Figure 2). The values represent normalized overlap frequencies, indicating the proportion of patients with one subtype who also exhibit another subtype.

The highest degree of overlap was observed between gLP and oLP, with 41% of patients with gLP also presenting oral manifestations. Similarly, a substantial overlap was found between gLP and cLP (35%).

Moderate overlap was observed between oLP and cLP, with approximately 29% of oral cases also showing skin involvement and 28% of cLP presenting oral manifestations.

In contrast, LPP demonstrated minimal overlap with other subtypes, with frequencies ranging from 2% to 8%, suggesting a largely distinct clinical presentation.

### 3.3. Comorbidities Across LP Subtypes

**Figure 3:**
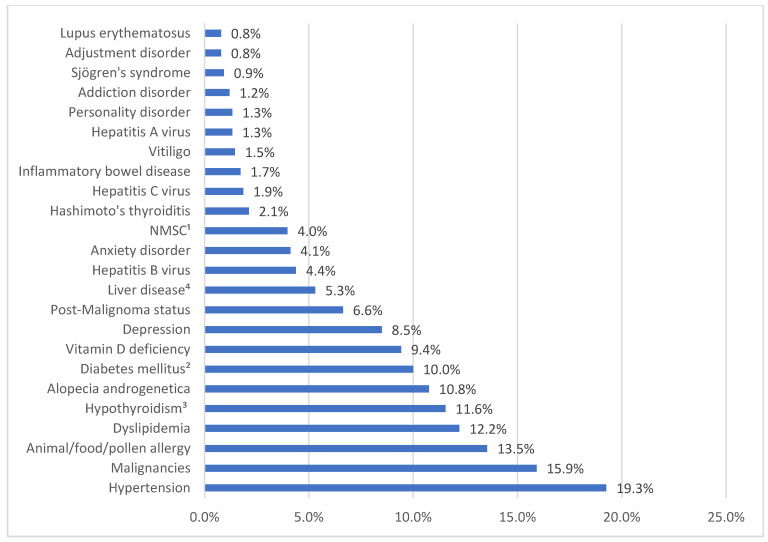
Comorbidities of LP patients (n = 754). Notes: ^1^ Non-melanoma skin cancer. ^2^ Criteria for hypothyroidism were defined as: (a) a documented diagnosis, (b) the presence of Hashimoto thyroiditis, or (c) surgical removal of the thyroid gland. ^3^ Includes type 1 and type 2 diabetes mellitus. ^4^ Includes alcoholic and metabolic liver damage. Additional comorbidities not illustrated above included: iIron deficiency (5.2%), anxiety disorder (4.1%), renal failure (2.9%), vitamin B deficiency (2.8%), polyneuropathy (2.4%), gastroesophageal reflux disease (2.4%), goiter (1.5%), fatigue (1.5%), HIV infection (0.9%), adjustment disorder (0.8%), ankylosing spondylitis (0.7%), cytomegalovirus infection (0.5%), somatization disorder (0.4%), eating disorder (0.3%), human papillomavirus infection (0.1%), and obsessive–compulsive disorder (0.1%).

Patients were systematically screened for comorbidities to assess potential associations with the different LP subtypes. The common comorbid conditions in patients with LP are presented in Figure 3 and Supplementary Table S1. Malignancies were more common in the cLP group (23.4%) than in other LP subtypes (oLP 14.9%, gLP 15.5%, LPP 12.0%). Hypothyroidism was most commonly found in patients with oLP (12.2%), LPP (12.0%), and cLP (10.6%) and was less common in the gLP subtype (7.3%). Hepatitis B- and C infections were uncommon across all LP subtypes. Hepatitis C infection was most frequent in patients with cLP (3.1%), followed by oLP and gLP (2.1% each), and was rare in LPP (0.3%). Hepatitis B infection showed a similar distribution, occurring in 7.1% of cLP, 2.4% of oLP, 2.1% of LPP, and 1.0% of gLP cases. Depression had the highest prevalence in the gLP group (11.6%), followed by oLP (8.2%), cLP (4.3%), and LPP (2.7%) (see Figure 1). Diabetes mellitus was more prevalent in patients with cLP (15.7%) compared to those with oLP (8.2%), gLP (7.3%), and LPP (5.5%).

### 3.4. Treatment Patterns and Clinical Response

Overall, 75.7% of patients received topical glucocorticoids, whereas oral glucocorticoids were administered in 8.9% of cases (see Table 2). Treatment with potent and very potent glucocorticoids (class III–IV) resulted in marked improvement or complete remission in over 40% of patients with cLP. In contrast, among patients with LPP, these agents achieved comparable improvement in only 4.6–7.1% of cases. Topical calcineurin inhibitors led to notable clinical improvement in gLP (41.3%) and cLP (36.8%) but yielded lower response rates in oLP (18.2%) and LPP (5.9%). Oral systemic glucocorticoids produced improvement in 20.8% of cLP and 21.9% of oLP cases but showed no clinical benefit in gLP (0.0%) and only minimal benefit in LPP (2.7%). Acitretin induced a marked clinical response in cLP (35.7%), whereas isotretinoin was effective in oLP (25.0%). In contrast, apremilast demonstrated no relevant clinical benefit across LP subtypes (see Table 3).

### 3.5. Exploratory Sensitivity and Adjusted Comorbidity Analyses

To further explore subtype-specific comorbidity patterns, we first performed a sensitivity analysis restricted to patients with a single major LP subtype (Supplementary Table S2), allowing comparison between mutually exclusive groups. In this analysis, 620 of 754 patients (82.2%) were included, comprising 162 cLP-only, 148 oLP-only, 39 gLP-only, and 271 LPP-only cases.

Age differed significantly across the mutually exclusive groups (Kruskal–Wallis *p* < 0.001), with LPP-only patients being younger compared to cLP-only and oLP-only patients. Sex distribution also varied significantly between groups (*p* < 0.001), with a marked female predominance in LPP-only patients and a male predominance in gLP-only patients.

Malignancies were most frequent in cLP-only patients (29.0%) compared to oLP-only (18.9%), gLP-only (15.4%), and LPP-only patients (14.4%) (*p* = 0.002). Similarly, diabetes mellitus was more prevalent in cLP-only patients (21.6%) compared to the other groups (*p* < 0.001). In contrast, no statistically significant differences were observed for hypothyroidism, depression, hepatitis B, or hepatitis C across the mutually exclusive subtypes (see Supplementary Table S2).

To avoid potential confounding factors such as differences in demographic characteristics and baseline comorbidity burden between subtype groups, we performed additional exploratory multivariable analyses (Supplementary Tables S3 and S4). Comorbidity burden was quantified using a modified Charlson Comorbidity Index (mCCI), with significant differences observed between mutually exclusive subtype groups for both mCCI (median 0 [IQR 0–2] in cLP-only vs. 0 [0–1] in oLP-only/gLP-only vs. 0 [0–0] in LPP-only; Kruskal–Wallis *p* < 0.001) and age-adjusted mCCI (median 2 [1–4] in cLP-only, 2 [1–3] in oLP-only, 2 [0–3] in gLP-only, and 1 [0–3] in LPP-only; *p* < 0.001), while the overall comorbidity count also differed significantly between groups (*p* = 0.022) (Supplementary Table S4). Direct pairwise multivariable logistic regression models were then performed between mutually exclusive LP subtype groups, adjusting for age, sex, and outcome-specific modified comorbidity burden.

In these adjusted analyses, the higher prevalence of malignancy in cLP-only patients remained significant compared with oLP-only patients (adjusted OR 2.70, 95% CI 1.45–5.04; *p* = 0.002) and LPP-only patients (adjusted OR 1.88, 95% CI 1.07–3.31; *p* = 0.028), with borderline significance compared with gLP-only patients (adjusted OR 2.71, 95% CI 1.00–7.35; *p* = 0.050). Similarly, diabetes mellitus remained significantly more frequent in cLP-only patients compared with oLP-only (adjusted OR 2.08, 95% CI 1.04–4.13; *p* = 0.037), gLP-only (adjusted OR 4.03, 95% CI 1.07–15.13; *p* = 0.039), and LPP-only patients (adjusted OR 3.26, 95% CI 1.62–6.56; *p* < 0.001). In contrast, no statistically significant adjusted differences were observed for hypothyroidism, depression, hepatitis B, or hepatitis C (all *p* > 0.05), although sparse-event estimates should be interpreted cautiously. These findings suggest that while some subtype-specific comorbidity patterns persisted after adjustment, part of the apparent variation across LP subtypes may be explained by differences in demographic characteristics and baseline comorbidity burden rather than subtype-specific effects alone. Overall, several subtype-specific patterns remained consistent, whereas others were attenuated after exclusion of overlapping cases.

## 4. Discussion

We reviewed 754 patients with LP treated at a tertiary care center with respect to comorbidities, anatomical involvement, and treatment outcomes. The most common subtypes—cLP, oLP, gLP, and LPP—occurred predominantly in middle-aged individuals (mean ages: 54.9, 57.1, 53.4, and 51.3 years, respectively), consistent with findings from previous studies [46,47,48].

All LP subtypes, except for genital LP—which showed a male predominance (57.7%)—demonstrated a female predominance in our cohort, with this being true for cLP (53.9%), oLP (59.1%), and LPP (83.2%), consistent with previous studies [6,46,47,48,49,50]. One possible explanation is that a substantial proportion of female patients may have received their diagnosis from a gynecologist and were therefore not fully captured in our dataset. In men, the glans penis is most affected [51] and typically easier to recognize. In women, however, the diagnosis can be more challenging, as the clinical and histopathological features may overlap with those of lichen sclerosus [52].

The overlap patterns observed in our study suggest that LP subtypes are not independent entities but part of a structured disease spectrum. The strongest overlap was observed between mucosal subtypes, with 41% of patients with gLP also presenting oLP, and a substantial overlap between gLP and cLP (35%). Moderate overlap was observed between oLP and cLP (approximately 29% and 28%). In contrast, the minimal overlap of LPP (2–8%) reinforces its role as a distinct follicular variant.

From a pathophysiological perspective, several of the observed comorbidity patterns may be biologically plausible. LP is considered a T-cell-mediated inflammatory disease characterized by cytotoxic damage to basal keratinocytes, with the involvement of IFN-γ, TNF-α, IL-2, and other proinflammatory pathways. Such chronic immune activation may partly explain reported links between LP and autoimmune disorders, thyroid disease, viral hepatitis, and psychological comorbidity. In addition, persistent epithelial inflammation and barrier disruption, particularly in mucosal disease, may contribute to malignant transformation in selected anatomical sites. However, these mechanisms are likely heterogeneous across LP subtypes, and our retrospective design does not allow conclusions regarding causality.

In the present study, we investigated the association between different clinical manifestations of lichen planus and selected comorbidities, including hepatitis B, hepatitis C, malignancies, Hashimoto’s thyroiditis and Vitiligo. In this registry-based cohort of patients with LP, no consistent associations were observed between LP subtypes and most investigated comorbidities. However, cLP was associated with malignancies, although the effect size was modest. Overall, a substantial proportion of the cohort had a concurrent cancer diagnosis at the time of LP diagnosis,, with rates varying by subtype: cutaneous LP (cLP) 23.4%, oral LP (oLP) 14.6%, genital LP (gLP) 15.5%, and LPP (12.0%). This finding is in line with a retrospective study from Finland, which reported a cancer prevalence of 19.06% among patients with cLP at the time of diagnosis [46]. The observed association between cLP and malignancies may have several explanations. Chronic inflammation has been proposed as a potential contributing factor in carcinogenesis [53], but the association observed in this study may also reflect confounding factors, particularly age and comorbidity burden.

Malignancies were more frequently documented among patients with cLP in the descriptive full-cohort analysis and in the mutually exclusive cLP-only group. After adjustment for age, sex, and outcome-specific modified CCI, cLP-only patients showed higher odds of malignancy compared with oLP-only patients (adjusted OR 2.70, 95% CI 1.45–5.04; *p* = 0.002) and LPP-only patients (adjusted OR 1.88, 95% CI 1.07–3.31; *p* = 0.028), with borderline significance compared with gLP-only patients (adjusted OR 2.71, 95% CI 1.00–7.35; *p* = 0.050). However, the observed effect sizes were modest to moderate, and the comparison with gLP-only was based on a small subgroup. This finding should therefore be interpreted cautiously and does not establish a causal or paraneoplastic relationship. Referral patterns, surveillance intensity, age structure, and residual confounding may all contribute to the observed differences.

Importantly, malignancies were analyzed as a composite outcome, which limits the ability to draw conclusions regarding site-specific cancer risk. Therefore, the observed association should be interpreted cautiously and does not imply causality.

According to the Swiss Cancer Study of the National Cancer Data Set—which excluded patients with non-melanoma skin cancer—the prevalence of cancer in the general Swiss population is approximately 2.4%, with individuals aged over 70 years comprising about 23–25% of the population [54]. In comparison, the cancer prevalence in our study population, which has a mean age of 53.13, appears markedly higher. This finding is noteworthy when compared to other facultative and obligate paraneoplastic dermatoses, such as dermatomyositis (14.8%) [55], pemphigus vulgaris (8%) [56], and acanthosis nigricans (55–61%) [57]. These findings raise the hypothesis that a subset of LP cases may be associated with underlying malignancy; however, causal relationships cannot be inferred from this retrospective analysis. Further prospective studies are required to clarify this association and to determine whether targeted malignancy screening may be beneficial in selected patients.

Similarly, the higher prevalence of diabetes mellitus observed in patients with cLP may be biologically plausible. Chronic low-grade systemic inflammation and shared metabolic risk factors have been proposed as potential links between inflammatory dermatoses and insulin resistance. However, given the retrospective design and lack of adjustment for confounding variables such as age and body mass index, this association should be interpreted with caution.

No statistically significant associations were identified, e.g., for hepatitis B and hepatitis C, or vitiligo. However, these findings should be interpreted with caution, as the number of events was low, resulting in limited statistical power and wide confidence intervals.

This is further supported by the sensitivity analysis of mutually exclusive subtypes, in which several associations were attenuated after the exclusion of overlapping cases.

Psychiatric disorders such as depression and anxiety have been reported in association with LP, particularly oLP [58,59,60], in several studies. In our cohort, the highest prevalence of depression was observed in the gLP group (11.6%), while anxiety was most commonly reported in patients with LPP (4.1%). However, the prevalence of depression and anxiety observed in our study was lower than the 27% and 28% reported in the current meta-analysis [61]. This discrepancy may be attributed to differences in study design, as the meta-analysis focused exclusively on oral LP, which may present with a higher psychological burden [61].

There is growing evidence of a possible link between cLP and oLP and thyroid disease, particularly hypothyroidism [62]. Several studies have reported an increased risk of thyroid dysfunction in patients with cLP, oLP and LPP, with hypothyroidism being the most frequently observed condition [28,63,64]. In our study, the prevalence of thyroid disease was 10.6% in patients with cLP, 12.2% with oLP, and 12.0% with LPP, which is notably lower than in the aforementioned studies. No statistically significant association was identified for Hashimoto’s thyroiditis, likely due to the low number of observed cases.

However, the association remains controversial, as other studies have failed to demonstrate a significant correlation between LP and thyroid disease [65].

The comparatively low number of documented autoimmune comorbidities in our cohort should also be interpreted with caution. First, common chronic disorders that are highly prevalent in the general population may be more readily documented in routine clinical care than less frequent autoimmune diseases, which may lead to differential ascertainment. Second, this was a retrospective hospital-based study, and comorbidities were captured from available records rather than through systematic screening. As a result, underreporting of less prominent or externally managed autoimmune conditions is possible. More generally, background prevalence of common age-related comorbidities may influence the apparent comorbidity profile and should be considered when interpreting associations in LP.

Hepatitis screening in LP remains controversial [19]. In our study, hepatitis B (7.1%) and hepatitis C (3.1%) were most frequently detected in patients with cLP, suggesting that subtype-specific screening strategies may be more appropriate than universal screening.

Among patients with LPP, the prevalence of coexisting cLP, gLP, and oLP was low (2–8%). In contrast, co-occurrence between mucosal and cutaneous subtypes was frequent, with approximately 41% of patients with gLP also exhibiting oLP and 35% cLP, while oLP and cLP overlapped in approximately 29% and 28% of cases, respectively.

The gene expression and cellular composition differ across LP subtypes and the LP, which is reflected clinically, as cLP, oLP, and gLP share several clinical similarities [66].

Potent and very potent topical glucocorticoids demonstrated good efficacy in patients with cLP and gLP (41.3% and 46.8%, respectively), whereas treatment responses were limited in LPP (4.6%). The management of LPP remains particularly challenging, as no causal therapy is currently available and treatment primarily aims to halt disease progression rather than achieve remission. Consequently, escalation to second- or third-line therapies is often required; however, these approaches frequently result in disease stabilization rather than significant improvement.

Topical calcineurin inhibitors showed the highest efficacy in gLP, likely due to enhanced penetration through genital mucosa compared with keratinized skin. Acitretin and isotretinoin demonstrated variable efficacy across LP subtypes. Acitretin was associated with high rates of clinical improvement in cLP, while isotretinoin showed favorable responses in oLP. Randomized controlled trials have confirmed the efficacy of acitretin in both oral and cutaneous LP, with significant rates of improvement and remission reported [36,67]. In contrast, evidence supporting isotretinoin remains limited to small studies and case series, primarily involving topical formulations for oral lesions or low-dose systemic therapy in lichen planus pigmentosus [68].

One limitation in the evaluation of treatment outcomes is that some patients received multiple therapeutic agents, which limits the ability to attribute clinical response to a single intervention.

In addition, we did not systematically assess concomitant medications used for comorbidities. This is relevant because drug-induced lichenoid eruptions and possibly LPP-like reactions may mimic or modify the clinical phenotype, introducing potential misclassification or confounding.

To further address the impact of subtype overlap, we conducted an exploratory sensitivity analysis restricted to patients with a single major LP subtype. While this approach allowed for statistically independent group comparisons, it required exclusion of patients with overlapping phenotypes, who represent a clinically relevant proportion of the LP population.

In this subset, some subtype-specific patterns, particularly the higher prevalence of malignancies and diabetes mellitus in cLP-only patients, remained consistent. However, other differences were attenuated, suggesting that overlap between subtypes may influence observed associations in the full cohort.

These findings highlight the complex and overlapping nature of LP manifestations. The pairwise multivariable logistic regression analyses were performed as exploratory sensitivity analyses in mutually exclusive subtype groups to partially address subtype overlap and potential confounding. Given the reduced sample size after exclusion of overlapping phenotypes, the low event numbers for several outcomes, and the residual confounding inherent to retrospective data, these adjusted findings should be interpreted as hypothesis-generating rather than confirmatory. As overlapping phenotypes are a key clinical feature of LP, analyses restricted to mutually exclusive subtypes should be regarded as complementary to the full-cohort descriptive analyses rather than representative of the overall disease spectrum.

## 5. Conclusions

In summary, this study represents the first large-scale comparative analysis of multiple LP subtypes within a single histopathologically confirmed cohort. Our findings indicate that the typical age of onset across LP subtypes is in the fifth decade of life and that nearly one in five patients had a concurrent malignancy at the time of diagnosis. Treatment responses varied substantially by subtype, with notable differences observed for topical glucocorticoids, calcineurin inhibitors, and systemic retinoids. Additional exploratory adjusted analyses in mutually exclusive subtype groups suggested that some observed comorbidity differences, particularly for malignancy and diabetes mellitus in cLP-only patients, persisted after adjustment for age, sex, and comorbidity burden, whereas several other observed differences were attenuated after adjustment. These exploratory findings should be interpreted cautiously and do not imply causality.

The major strengths of this study include the large sample size, the systematic integration of clinical and histopathologic data, and the inclusion of multiple LP variants, allowing for a comprehensive evaluation of comorbidity patterns and treatment approaches. However, several limitations must be acknowledged. First, despite the overall cohort size, the sample size of individual LP subtypes—particularly genital LP, nail LP, ocular LP, and some comorbidity subgroups—was limited, which reduced statistical power and may have contributed to unstable estimates and wide confidence intervals. This also limited the ability to detect more subtle subtype-specific associations. Second, formal statistical testing of the main subtype comparisons was limited due to overlapping phenotypes; however, an additional exploratory sensitivity analysis using mutually exclusive subtypes was performed. This required the exclusion of overlapping cases and further reduced the available sample size. To further address potential confounding, we additionally performed exploratory pairwise multivariable logistic regression analyses in mutually exclusive subtype groups using adjustment for age, sex, and modified comorbidity burden; however, these analyses remain exploratory and are limited by residual confounding, low event numbers for some outcomes, and the incomplete availability of all Charlson Comorbidity Index domains. Third, the retrospective design may have resulted in the incomplete documentation of clinical characteristics and comorbidities. Furthermore, data from general practitioners, dermatologists in private practice, and dentists were not captured, potentially leading to the underrepresentation of patients with milder disease or isolated oral LP. In addition, concomitant medications that may have contributed to lichenoid eruptions were not systematically available and could not be incorporated into the adjusted analyses.

## Figures and Tables

**Table 1:** Demographic and clinical characteristics of patients with LP subtypes (n = 754).

	cLP (n = 256)	oLP (n = 247)	gLP (n = 97)	LPP (n = 291)
Age in years, mean (SD)	54.94 (16.63)	57.10 (15.70)	53.41 (16.03)	51.34 (16.81)
Male, n (%)	46.1%	40.9%	58.8%	16.8%
Female, n (%)	53.9%	59.1%	41.2%	83.2%
Number of involved anatomical sites, n (%)				
1	71 (27.7%)	135 (54.7%)	37 (38.1%)	282 (86.8%)
2	48 (18.8%)	40 (16.2%)	24 (24.7%)	16 (4.9%)
3	63 (24.6%)	29 (11.7%)	14 (14.4%)	11 (3.4%)
4	30 (11.7%)	19 (7.7%)	10 (10.3%)	4 (1.2%)
5	18 (7.0%)	9 (3.9%)	4 (4.1%)	2 (0.6%)
6	9 (3.5%)	6 (2.4%)	3 (3.1%)	5 (1.5%)
7	7 (2.7%)	4 (1.6%)	3 (3.1%)	2 (0.6%)
8	3 (1.2%)	2 (0.8%)	1 (1.0%)	3 (0.9%)
9	4 (1.6%)	2 (0.6%)	0 (0.0%)	0 (0.0%)
10	2 (0.8%)	1 (0.4%)	1 (1.0%)	0 (0.0%)
11	0 (0.0%)	0 (0.0%)	0 (0.0%)	0 (0.0%)
12	1 (0.2%)	0 (0.0%)	0 (0.0%)	0 (0.0%)

Note: LP = lichen planus; LPP = lichen planopilaris; gLP = genital lichen planus; oLP = oral lichen planus; cLP = cutaneous lichen planus. Some patients were classified under more than one LP subtype.

**Table 2:** Overview of therapeutic approaches categorized by LP subtype (n = 754).

	cLP (n = 256), (%)	oLP (n = 247), (%)	gLP (n = 97), (%)	LPP (n = 291), (%)
**Topical glucocorticoids**				
Mild (I)	1 (0.4%)			
Moderate (II)	6 (2.3%)		4 (4.1%)	
Potent (III)	150 (58.6%)		47 (48.5%)	140 (48.1%)
Very potent (IV)	101 (39.5%)		41 (42.3%)	219 (75.3%)
**Corticosteroid dental paste**		144 (58.3%)		
**Steroidal mouthwash**		130 (52.6%)		
**Topical calcineurin inhibitors**	19 (7.4%)	11 (4.5%)	46 (47.4%)	34 (11.7%)
**Minoxidil**				124 (42.6%)
**Systemic therapy**				
Oral steroids	48 (18.8%)	54 (21.9%)	14 (14.4%)	37 (12.7%)
Intralesional steroids	4 (1.6%)			4 (1.4%)
Acitretin	14 (5.5%)	18 (7.3%)	9 (9.3%)	18 (6.2%)
Isotretinoin	4 (1.6%)	20 (8.1%)	6 (6.2%)	13 (4.5%)
Hydroxychloroquine		17 (6.9%)		111 (38.1%)
Apremilast	7 (2.7%)	22 (8.9%)	7 (7.2%)	
**UV Therapy**	21 (8.2%)		1 (1.0%)	
**No treatment**	18 (7.0%)	19 (7.7%)		
**Excision**			5 (5.2%)	

Note: LP = lichen planus; LPP = lichen planopilaris; gLP = genital lichen planus; oLP = oral lichen planus; cLP = cutaneous lichen planus. Therapeutic agents may have been used in combination with other treatments. Additional therapeutics not illustrated above included methotrexate, azathioprine, mycophenolate mofetil, cyclosporin, doxycycline, tetracycline, IL-17A inhibitors, and JAKinhibitors.

**Table 3:** Therapy outcome of LP subtypes.

	Therapy Outcome of cLP (n = 256)	Therapy Outcome of oLP (n = 247)	Therapy Outcome of gLP (n = 97)	Therapy Outcome of LPP (n = 291)
Topical glucocorticoids				
Potent (III)				
0	31 (20.7%)		8 (17.0%)	49 (35.0%)
1	46 (30.7%)		15 (31.9%)	70 (50.0%)
2	62 (41.3%)		22 (46.8%)	10 (7.1%)
3	11 (7.3%)		2 (4.9%)	11 (7.9%)
Very potent (IV)				
0	12 (11.9%)		8 (19.5%)	55 (25.1%)
1	41 (40.6%)		15 (36.6%)	146 (66.7%)
2	43 (42.6%)		16 (39.0%)	10 (4.6%)
3	5 (5.0%)		2 (4.9%)	8 (3.7%)
Corticosteroid dental paste				
0		47 (32.6%)		
1		52 (36.1%)		
2		40 (27.8%)		
3		5 (3.5%)		
Steroidal mouthwash				
0		35 (26.9%)		
1		53 (40.8%)		
2		35 (26.9%)		
3		7 (5.4%)		
Topical calcineurin inhibitors				
0	4 (21.1%)	4 (36.4%)	6 (13.0%)	9 (26.5%)
1	6 (31.6%)	4 (36.4%)	15 (32.6%)	22 (64.7%)
2	7 (36.8%)	2 (18.2%)	19 (41.3%)	2 (5.9%)
3	2 (10.5%)	1 (9.1%)	6 (13.0%)	1 (2.9%)
Minoxidil				
0				26 (21.0%)
1				88 (71.0%)
2				3 (2.4%)
3				7 (5.6%)
Oral steroids				
0	7 (14.6%)	7 (21.9%)	2 (14.3%)	14 (37.8%)
1	27 (56.3%)	15 (46.9%)	12 (85.7%)	20 (54.1%)
2	10 (20.8%)	7 (21.9%)	0 (0.0%)	1 (2.7%)
3	4 (8.3%)	3 (9.4%)	0 (0.0%)	2 (5.4%)
Acitretin				
0	6 (42.9%)	4 (22.2%)	3 (33.3%)	8 (44.4%)
1	1 (7.1%)	11 (61.1%)	4 (44.4%)	9 (50.0%)
2	5 (35.7%)	1 (5.6%)	1 (11.1%)	0 (0.0%)
3	2 (14.3%)	2 (11.1%)	1 (11.1%)	1 (5.6%)
Isotretinoin				
0	2 (50.0%)	6 (30.0%)	3 (50.0%)	4 (30.8%)
1	1 (25.0%)	8 (40.0%)	3 (50.0%)	8 (61.5%)
2	1 (25.0%)	5 (25.0%)	0 (0.0%)	0 (0.0%)
3	0 (0.0%)	1 (5.0%)	0 (0.0%)	1 (7.7%)
Hydroxychloroquine				
0		1 (5.9%)		22 (19.8%)
1		13 (76.5%)		71 (64.0%)
2		1 (5.9%)		2 (1.8%)
3		2 (11.8%)		16 (14.4%)
Apremilast				
0	0 (0.0%)	0 (0.0%)	0 (0.0%)	
1	6 (85.7%)	19 (86.4%)	7 (100.0%)	
2	0 (0.0%)	1 (4.5%)	0 (0.0%)	
3	1 (14.3%)	2 (9.1%)	0 (0.0%)	
UV therapy				
0	5 (23.8%)		0 (0.0%)	
1	13 (61.9%)		1 (100.0%)	
2	1 (4.8%)		0 (0.0%)	
3	2 (9.5%)		0 (0.0%)	

Note: Other therapeutic agents not listed: glucocorticoids class I (n = 1), glucocorticoids class II (n = 10), methotrexate (n = 10), cyclosporine (n = 6), JAKinhibitors (n = 6), IL-17 inhibitors (n = 7), doxycycline (n = 4), tetracycline (n = 2), and excision (n = 5). Legend: 0: No improvement/progression of the disease. 1: little improvement/stable response. 2: Strong improvement/complete remission. 3: Loss of follow-up.

## Data Availability

The datasets generated and analyzed during the current study are not publicly available due to privacy and ethical considerations. However, they are available from the corresponding author upon reasonable request.

## Supplementary Materials

The following supporting information can be downloaded at https://www.mdpi.com/article/10.3390/jcm15114101/s1. Table S1: Proportion of comorbidities in LP subtypes; Table S2: Sensitivity analysis of mutually exclusive LP subtypes (pure groups); Table S3: Pairwise exploratory multivariable logistic regression models in mutually exclusive LP subtypes. Table S4: Modified Charlson Comorbidity Index and comorbidity burden by subtype. Figure S1: Skin locations (%) in 256 cLP patients; Figure S2: Skin locations (%) in 247 oLP patients; Figure S3: Skin locations (%) in 97 gLP patients; Figure S4: Skin locations (%) in 291 LPP patients.

## Abbreviations

The following abbreviations are used in this manuscript:

LP	Lichen planus
cLP	Cutaneous lichen planus
oLP	Oral lichen planus
gLP	Genital lichen planus
LPP	Lichen planopilaris
NMSC	Non-melanoma skin cancer
