# Supplementary material for: Evaluation of Comorbidities and Treatment Outcome in Various Subtypes of Lichen Planus: A Single-Center Retrospective Study
Source: J Clin Med. 2026 May 26;15(11):4101. doi: 10.3390/jcm15114101 (PMC13258672; doi:10.3390/jcm15114101)
Supplement: Supplementary file 1 [file jcm-15-04101-s001.zip › LP_Supplemental_Figure 2.pdf]

Supplemental Figure S2: Skin localization (%) in 247 oLP patients

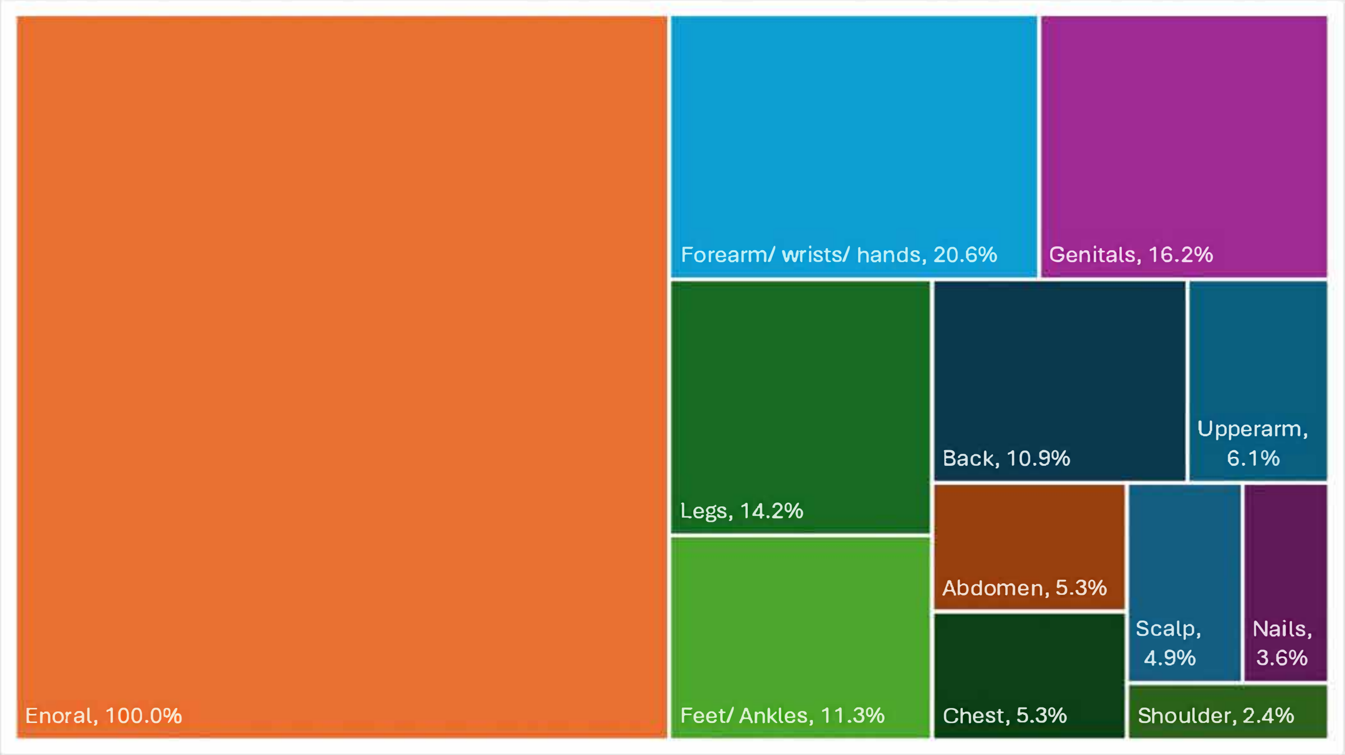

Note:
Additional skin localizations not represented in the tree map included:
Esophagus (1.6%), face (1.6%), anal/ perianal region (1.6%), axilla (1.2%), gluteal (1.2%), neck (0.8.%), submammary (0.4%), ocular (0.4%), nasal (0.4%
