# Supplementary material for: Evaluation of Comorbidities and Treatment Outcome in Various Subtypes of Lichen Planus: A Single-Center Retrospective Study
Source: J Clin Med. 2026 May 26;15(11):4101. doi: 10.3390/jcm15114101 (PMC13258672; doi:10.3390/jcm15114101)
Supplement: Supplementary file 1 [file jcm-15-04101-s001.zip › LP_Supplemental_Figure 4.pdf]

Supplemental Figure 4: Skin localization (%) in 291 LPP patients

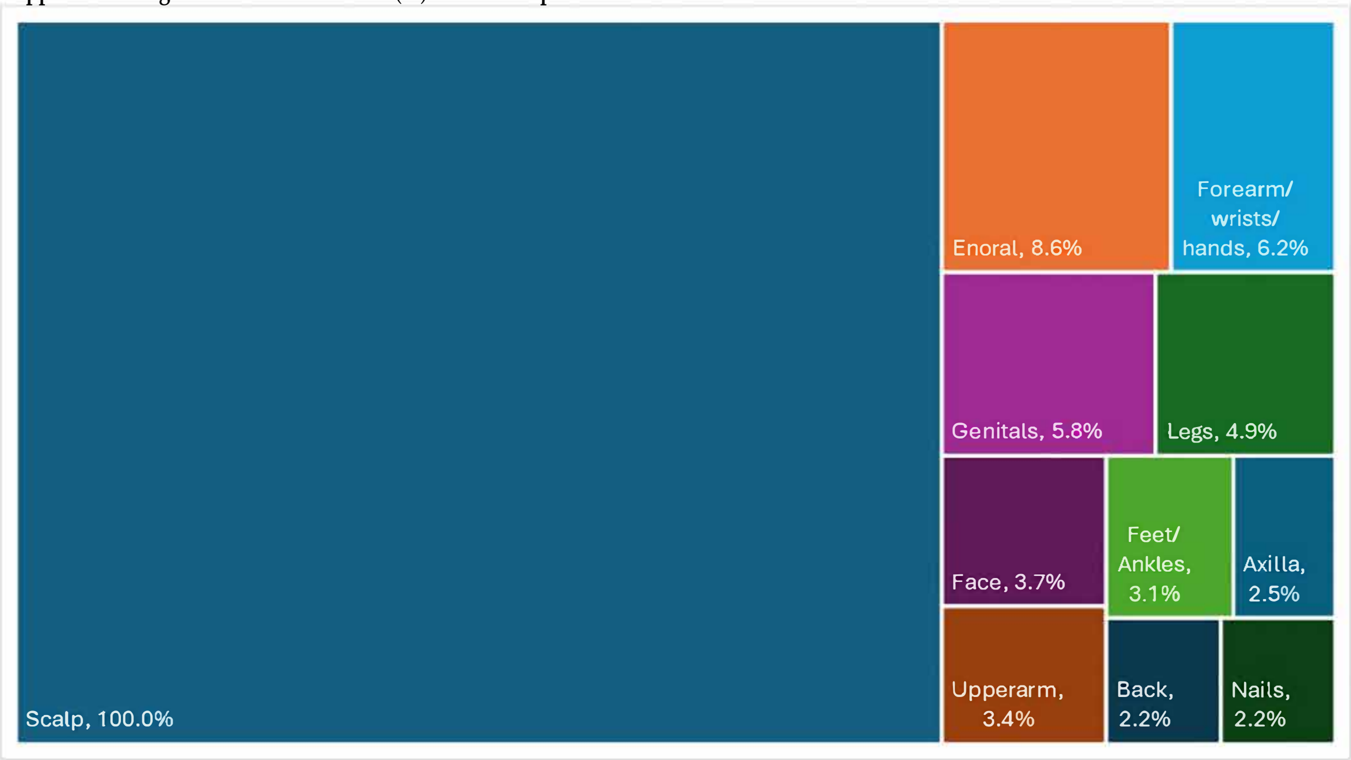

Note:
Additional skin localizations not represented in the tree map included:
Neck (0.9%), chest (0.6%), inguinal (0.3%), anal/perianal region (0.3%) and eosophagus (0.3%).
