# Supplementary material for: Evaluation of Comorbidities and Treatment Outcome in Various Subtypes of Lichen Planus: A Single-Center Retrospective Study
Source: J Clin Med. 2026 May 26;15(11):4101. doi: 10.3390/jcm15114101 (PMC13258672; doi:10.3390/jcm15114101)
Supplement: Supplementary file 1 [file jcm-15-04101-s001.zip › LP_Supplemental_Table S2.pdf]

Table S2. Sensitivity analysis of mutually exclusive LP subtypes (pure groups).

	cLP only (n=162)	oLP only (n=148)	gLP only (n=39)	LPP only (n=271)	p-value
**Age in years, mean (SD)**	55.8 (16.3)	58.1 (15.2)	55.5 (14.5)	50.5 (16.5)	<0.001*
**Female sex, n (%)**	86 (53.1%)	83 (56.1%)	12 (30.8%)	228 (84.1%)	<0.001**
**Comorbidities, n (%)**					
Malignancies	47 (29.0%)	28 (18.9%)	6 (15.4%)	39 (14.4%)	0.002**
Diabetes mellitus	35 (21.6%)	15 (10.1%)	3 (7.7%)	15 (5.5%)	<0.001**
Hypothyroidism	21 (13.0%)	21 (14.2%)	1 (2.6%)	32 (11.8%)	0.253**
Depression	13 (8.0%)	12 (8.1%)	4 (10.3%)	22 (8.1%)	0.973**
Hepatitis B	9 (5.6%)	7 (4.7%)	1 (2.6%)	5 (1.8%)	0.178**
Hepatitis C	5 (3.1%)	3 (2.0%)	2 (5.1%)	1 (0.4%)	0.065**

*Kruskal–Wallis test
** Chi-square test (or Fisher's exact test where appropriate)

Note: This analysis was restricted to patients with a single major LP subtype (mutually exclusive groups). Patients with overlapping subtype involvement were excluded. Results are considered exploratory due to reduced sample size and exclusion of clinically relevant overlap.
