# Supplementary material for: Evaluation of Comorbidities and Treatment Outcome in Various Subtypes of Lichen Planus: A Single-Center Retrospective Study
Source: J Clin Med. 2026 May 26;15(11):4101. doi: 10.3390/jcm15114101 (PMC13258672; doi:10.3390/jcm15114101)
Supplement: Supplementary file 1 [file jcm-15-04101-s001.zip › LP_Supplemental_Table S3.pdf]

Table S3. Pairwise exploratory multivariable logistic regression models in mutually exclusive LP subtypes. Each row reports a direct pairwise comparison between two mutually exclusive LP subtype groups. Models were fitted separately for each pair and adjusted for age, male sex, and modified Charlson Comorbidity Index (mCCI). To avoid circular adjustment, the modeled outcome was excluded from the mCCI covariate when applicable, specifically for malignancy and diabetes mellitus. Results are odds ratios (ORs) with 95% confidence intervals. Rare-event estimates should be interpreted cautiously.

Outcome	Comparison	Events, n/N	Adjusted OR (95% CI)	p-value	Note
**Malignancy**	cLP-only vs oLP-only	44/162 vs 18/148	2.70 (1.45-5.04)	0.002	
**Malignancy**	cLP-only vs gLP-only	44/162 vs 6/39	2.71 (1.00-7.35)	0.050	
**Malignancy**	cLP-only vs LPP-only	44/162 vs 32/271	1.88 (1.07-3.31)	0.028	
**Malignancy**	oLP-only vs gLP-only	18/148 vs 6/39	0.70 (0.25-2.01)	0.512	
**Malignancy**	oLP-only vs LPP-only	18/148 vs 32/271	0.83 (0.43-1.63)	0.595	
**Malignancy**	gLP-only vs LPP-only	6/39 vs 32/271	0.94 (0.31-2.87)	0.908	
**Diabetes mellitus**	cLP-only vs oLP-only	35/162 vs 15/148	2.08 (1.04-4.13)	0.037	
**Diabetes mellitus**	cLP-only vs gLP-only	35/162 vs 3/39	4.03 (1.07-15.13)	0.039	
**Diabetes mellitus**	cLP-only vs LPP-only	35/162 vs 15/271	3.26 (1.62-6.56)	<0.001	
**Diabetes mellitus**	oLP-only vs gLP-only	15/148 vs 3/39	1.72 (0.43-6.90)	0.443	
**Diabetes mellitus**	oLP-only vs LPP-only	15/148 vs 15/271	1.68 (0.75-3.76)	0.208	
**Diabetes mellitus**	gLP-only vs LPP-only	3/39 vs 15/271	1.85 (0.39-8.82)	0.442	
**Hypothyroidism**	cLP-only vs oLP-only	21/162 vs 21/148	0.93 (0.47-1.83)	0.825	
**Hypothyroidism**	cLP-only vs gLP-only	21/162 vs 1/39	3.88 (0.49-30.99)	0.200	
**Hypothyroidism**	cLP-only vs LPP-only	21/162 vs 32/271	1.48 (0.78-2.79)	0.228	
**Hypothyroidism**	oLP-only vs gLP-only	21/148 vs 1/39	4.96 (0.63-38.89)	0.128	
**Hypothyroidism**	oLP-only vs LPP-only	21/148 vs 32/271	1.38 (0.73-2.61)	0.318	
**Hypothyroidism**	gLP-only vs LPP-only	1/39 vs 32/271	0.32 (0.04-2.61)	0.290	
**Depression**	cLP-only vs oLP-only	13/162 vs 12/148	0.98 (0.42-2.28)	0.959	
**Depression**	cLP-only vs gLP-only	13/162 vs 4/39	0.64 (0.19-2.18)	0.474	
**Depression**	cLP-only vs LPP-only	13/162 vs 22/271	1.10 (0.50-2.39)	0.817	
**Depression**	oLP-only vs gLP-only	12/148 vs 4/39	0.72 (0.21-2.46)	0.601	
**Depression**	oLP-only vs LPP-only	12/148 vs 22/271	1.13 (0.51-2.49)	0.767	

**Depression**	gLP-only vs LPP-only	4/39 vs 22/271	1.93 (0.54-6.87)	0.311	
**Hepatitis B**	cLP-only vs oLP-only	9/162 vs 7/148	1.05 (0.36-3.08)	0.924	
**Hepatitis B**	cLP-only vs gLP-only	9/162 vs 1/39	2.39 (0.28-20.04)	0.423	
**Hepatitis B**	cLP-only vs LPP-only	9/162 vs 5/271	3.05 (0.92-10.16)	0.069	
**Hepatitis B**	oLP-only vs gLP-only	7/148 vs 1/39	2.45 (0.27-22.27)	0.426	
**Hepatitis B**	oLP-only vs LPP-only	7/148 vs 5/271	2.66 (0.75-9.38)	0.128	
**Hepatitis B**	gLP-only vs LPP-only	1/39 vs 5/271	NA	NA	not estimable (sparse events/singular model)
**Hepatitis C**	cLP-only vs oLP-only	5/162 vs 3/148	1.42 (0.31-6.59)	0.654	
**Hepatitis C**	cLP-only vs gLP-only	5/162 vs 2/39	0.44 (0.07-2.57)	0.361	
**Hepatitis C**	cLP-only vs LPP-only	5/162 vs 1/271	8.39 (0.91-76.98)	0.060	
**Hepatitis C**	oLP-only vs gLP-only	3/148 vs 2/39	0.36 (0.05-2.49)	0.299	
**Hepatitis C**	oLP-only vs LPP-only	3/148 vs 1/271	2.64 (0.23-30.29)	0.435	
**Hepatitis C**	gLP-only vs LPP-only	2/39 vs 1/271	8.15 (0.50-131.94)	0.140	

Abbreviations: cLP, cutaneous lichen planus; oLP, oral lichen planus; gLP, genital lichen planus; LPP, lichen planopilaris; CCI, Charlson Comorbidity Index; mCCI, modified Charlson Comorbidity Index; OR, odds ratio; CI, confidence interval.
