# Supplementary material for: Evaluation of Comorbidities and Treatment Outcome in Various Subtypes of Lichen Planus: A Single-Center Retrospective Study
Source: J Clin Med. 2026 May 26;15(11):4101. doi: 10.3390/jcm15114101 (PMC13258672; doi:10.3390/jcm15114101)
Supplement: Supplementary file 1 [file jcm-15-04101-s001.zip › LP_Supplemental_Table S4.pdf]

Table S4. Modified Charlson Comorbidity Index and comorbidity burden by subtype

The modified CCI was calculated using available Charlson domains in the dataset: diabetes mellitus, liver disease, renal failure, connective tissue/rheumatologic disease, malignancy, and HIV infection. Because not all Charlson domains and severity levels were available, this score should not be interpreted as a complete Charlson Comorbidity Index. Age-adjusted mCCI additionally assigns age points according to standard Charlson age categories.

Subtype	n	Age, mean (SD)	Female, n (%)	mCCI, median (IQR)	Age-adjusted mCCI, median (IQR)	Comorbidity count, median (IQR)
**cLP-only**	162	55.8 (16.3)	86 (53.1%)	0 (0-2)	2 (1-4)	2 (1-4)
**oLP-only**	148	58.1 (15.2)	85 (57.4%)	0 (0-1)	2 (1-3)	2 (0-3)
**gLP-only**	39	55.5 (14.5)	12 (30.8%)	0 (0-1)	2 (0-3)	2 (1-3)
**LPP-only**	271	50.5 (16.5)	228 (84.1%)	0 (0-0)	1 (0-3)	2 (1-3)
**Kruskal-Wallis p-value**				<0.001	<0.001	0.022

Abbreviations: cLP, cutaneous lichen planus; oLP, oral lichen planus; gLP, genital lichen planus; LPP, lichen planopilaris; CCI, Charlson Comorbidity Index; mCCI, modified Charlson Comorbidity Index; OR, odds ratio; CI, confidence interval.
