# Supplementary material for: Evaluation of Comorbidities and Treatment Outcome in Various Subtypes of Lichen Planus: A Single-Center Retrospective Study
Source: J Clin Med. 2026 May 26;15(11):4101. doi: 10.3390/jcm15114101 (PMC13258672; doi:10.3390/jcm15114101)
Supplement: Supplementary file 1 [file jcm-15-04101-s001.zip › LP_Supplemental_table_S1.pdf]

Table S1. Proportion of comorbidities in LP subtypes (N=754)

cLP (N=256)	(%)	oLP (N=247)	(%)	LPP (N=291)	(%)	gLP (N=97)	(%)
Malignancies	23.4	Malignancies	14.6	Malignancies	12.0	Malignancies	13.1
Hypertension	22.7	Hypertension	21.2	Hypertension	13.1	Hypertension	16.7
Dyslipidemia	17.6	Dyslipidemia	13.1	Dyslipidemia	7.6	Dyslipidemia	12.5
Diabetes mellitus[3]	15.7	Diabetes mellitus[3]	8.2	Diabetes mellitus[3]	5.5	Diabetes mellitus[3]	7.3
Animal/ food/ pollen allergy	13.3	Animal/ food/ pollen allergy	16.3	Animal/ food/ pollen allergy	13.5	Animal/ food/ pollen	15.6
Hypothyroidism[2]	10.6	Hypothyroidism[2]	12.2	Hypothyroidism[2]	12.0	Hypothyroidism[2]	7.3
Vitamin D deficiency	10.2	Vitamin D deficiency	6.9	Vitamin D deficiency	11.3	Vitamin D deficiency	7.3
Depression	8.6	Depression	8.2	Depression	8.6	Depression	11.5
Liver disease[4]	7.5	Liver disease[4]	6.1	Liver disease[4]	4.5	Liver disease[4]	6.3
Hepatitis B virus	7.1	Hepatitis B virus	6.1	Hepatitis B virus	2.1	Hepatitis B virus	1.0
Post-malignoma status	5.1	Post-malignoma status	6.5	Post-malignoma status	7.6	Post-malignoma status	3.1
Alopecia androgenetica	5.1	Alopecia androgenetica	4.1	Alopecia androgenetica	20.6	Alopecia androgenetica	4.2
Anxiety disorder	4.3	Anxiety disorder	2.4	Anxiety disorder	4.5	Anxiety disorder	4.2
Inflammatory bowel disease	3.1	Inflammatory bowel disease	2.4	Inflammatory bowel disease	0.7	Inflammatory bowel disease	2.1
NMSC[1]	3.9	NMSC[1]	4.9	NMSC[1]	3.8	NMSC[1]	2.1
Hepatitis C virus	3.1	Hepatitis C virus	2.4	Hepatitis C virus	0.3	Hepatitis C virus	2.1
Addiction disorder	2.7	Addiction disorder	0.4	Addiction disorder	0.3	Addiction disorder	2.1
Hepatitis A virus	2.7	Hepatitis A virus	1.2	Hepatitis A virus	0.7	Hepatitis A virus	1.0
Hashimoto's thyroiditis	2.4	Hashimoto's thyroiditis	2.0	Hashimoto's thyroiditis	2.1	Hashimoto's thyroiditis	0.0
Vitiligo	2.0	Vitiligo	0.8	Vitiligo	1.0	Vitiligo	3.1
Personality disorder	1.6	Personality disorder	1.2	Personality disorder	1.0	Personality disorder	2.1
Adjustment disorder	1.2	Adjustment disorder	0.0	Adjustment disorder	1.0	Adjustment disorder	0.0
Lupus erythematodes	0.8	Lupus erythematosus	1.2	Lupus erythematodes	0.7%	Lupus erythematodes	0.0%
Sjögren's syndrome	0.4	Sjögren’s syndrome	1.6	Sjögren’s syndrome	1.0%	Sjögren’s syndrome	1.0%

Notes:
[1] Non-melanoma skin cancer
[2] Criteria for hypothyroidism were defined as: (a) a documented diagnosis, (b) the presence of Hashimoto thyroiditis, or (c) surgical removal of the thyroid gland.
[3] Includes type 1 and type 2 diabetes mellitus.
[4] Includes alcoholic liver damage and metabolic liver damage
